# Comparison of unilateral biportal endoscopic lumbar interbody fusion (UBE-LIF) and conventional open TLIF in degenerative lumbar spine disease: a radiological, clinical, and laboratory study

**DOI:** 10.1186/s13018-026-06946-6

**Published:** 2026-05-12

**Authors:** Yunus Şamil Gönüldenk, Mustafa Özyıldıran, Abdullah Merter

**Affiliations:** 1 Department of Orthopedics and Traumatology, Ankara University Faculty of Medicine, Ankara, Turkey; 2 Department of Orthopedics and Traumatology, Sandıklı State Hospital, Afyonkarahisar, Turkey

**Keywords:** Degenerative lumbar instability, Degenerative lumbar spine disease, Unilateral biportal endoscopy (UBE), Lumbar interbody fusion (LIF), UBE-LIF, Conventional open TLIF

## Abstract

**Background:**

Parallel to the advancement of endoscopic techniques, unilateral biportal endoscopic lumbar interbody fusion (UBE-LIF) has become a popular surgical method in the treatment of degenerative lumbar instability. This study aimed to compare the clinical and radiological outcomes of UBE-LIF and conventional open TLIF techniques.

**Methods:**

Patients with single-level degenerative lumbar spondylolisthesis who underwent either UBE-LIF or conventional open TLIF at our institution between January 2021 and May 2024 were included in this single-center study. Clinical outcomes were assessed using Visual analogue scale (VAS) scores, Japanese Orthopaedic Association (JOA) scores, and Oswestry Disability Index (ODI), both preoperatively and postoperatively. Operative time, length of hospital stay, laboratory markers of muscle injury (hemoglobin, CRP, ESR, WBC, myoglobin), and radiological fusion rates were also evaluated.

**Results:**

Among the 100 patients who met the inclusion criteria, UBE-LIF was performed in 74, and conventional open TLIF in 26. JOA, VAS-leg, VAS-low back pain, and ODI scores significantly improved in both groups postoperatively (*p* < 0.001). Clinical scores did not differ significantly between the groups at any time point (*p* > 0.05). Postoperative blood transfusion was required in 3 patients (11.5%) in the conventional group, whereas none was needed in the UBE-LIF group (*p* = 0.003). Length of hospital stay was also significantly shorter in the UBE-LIF group (45.4 vs. 98.6 h, *p* < 0.001). The difference in 12-month postoperative fusion rates between UBE-LIF (100%) and conventional open TLIF (92.3%) was not significant (*p* = 0.07). Postoperative progression of Goutallier grade (paraspinal muscle degeneration) was not observed in the UBE-LIF group, whereas it occurred in 21 patients (80.8%) in the conventional open TLIF group (*p* < 0.001).

**Conclusion:**

Clinical outcomes improved similarly with both techniques. However, postoperative muscle degeneration, intraoperative blood loss, and length of hospital stay were more favorable in the UBE-LIF group. UBE-LIF appears to be a strong alternative to conventional open TLIF.

**Supplementary Information:**

The online version contains supplementary material available at 10.1186/s13018-026-06946-6.

## Introduction

Lumbar degenerative disease is a common spinal disorder in adults, characterized by age-related degeneration of the lumbar spine [1–3]. It serves as a general term encompassing a spectrum of conditions that present with clinical manifestations such as low back pain, radiculopathy, and neurogenic claudication, which result from degenerative changes in the intervertebral discs, facet joints, articular cartilage, and ligaments [3–5]. Fusion surgery may be required in cases of lumbar degenerative disease with associated instability [2, 3, 6]. Lumbar interbody fusion (LIF) is a well-established surgical method to achieve decompression, provide spinal stability, and restore spinal function [3, 7, 8].

Multiple surgical approaches have been described over the years for lumbar interbody fusion, each with distinct advantages and limitations [3, 5, 7–9]. Interbody fusion was traditionally performed either via an anterior approach, known as anterior lumbar interbody fusion (ALIF), or via a posterior approach, known as posterior lumbar interbody fusion (PLIF) [7, 8]. To minimize paraspinal muscle dissection and reduce the risk of iatrogenic injury, alternative approaches have been developed over the years, including oblique lumbar interbody fusion (OLIF) and lateral lumbar interbody fusion (LLIF) [7–9]. One of the techniques developed as an alternative to PLIF is transforaminal lumbar interbody fusion (TLIF). Using a unilateral transforaminal approach, TLIF reduces retraction of the dural sac and nerve roots, which lowers the risk of root-related complications and has made it more popular than PLIF [2, 8, 10–14].

TLIF is an effective surgical technique for achieving spinal fusion and decompression in the treatment of lumbar degenerative diseases [10–14]. As an open surgical technique, conventional TLIF is associated with substantial soft tissue morbidity, potentially resulting in adverse clinical outcomes [2, 15–18]. In recent years, minimally invasive techniques become widely used in lumbar interbody fusion with the aim of reducing surgical trauma and paraspinal muscle injury, minimizing blood loss, and accelerating postoperative recovery [1, 2, 5, 6, 19].

Parallel to the advancement of endoscopic techniques, endoscopy has gained popularity in spine surgery, as in other surgical fields [20–23]. In recent years, unilateral biportal endoscopy (UBE) has emerged as a novel minimally invasive endoscopic spinal surgery technique and has been shown to be safe and effective [20–22]. UBE employs two portals, with the cranial portal for visualization and the caudal portal for working. This separation provides a geometric advantage by allowing independent movement of the endoscope and instruments, thereby enhancing maneuverability. The main advantages of UBE are the use of standard 4.0-mm rigid endoscopes, the absence of a need for tubular retractors or specialized channel-based systems, and the ability to employ conventional instruments such as Kerrison punches [21]. UBE has been effectively applied to treat a range of lumbar spine conditions, such as intervertebral disc herniation and decompression of spinal canal or foraminal stenosis [20–23]. In recent years, UBE has also been introduced for use in lumbar interbody fusion [1–3, 5, 6].

There are several studies reporting successful clinical outcomes of unilateral biportal endoscopic lumbar interbody fusion (UBE-LIF) [1–3, 5, 6, 19, 24, 25]. However, to the best of our knowledge, only a limited number of studies have compared UBE-LIF and conventional open TLIF [3, 6]. Therefore, this study aimed to compare the clinical and radiological outcomes of UBE-LIF and conventional open TLIF techniques.

## Methods

This study was a retrospective analysis of prospectively collected data from patients treated at our institution between January 2021 and May 2024. Patients with radiologically and clinically confirmed single-level degenerative lumbar spondylolisthesis who underwent either UBE-LIF or conventional open TLIF were included. This research was conducted following approval from the ethics committee of our institution (Decision number: i10-666-23). Written informed consent was obtained from the patients, and the study was carried out in accordance with the Declaration of Helsinki.

This research included patients who fulfilled all the following inclusion criteria: (1) Patients with single-level lumbar instability, defined as ≥ 10° of angular motion or ≥ 4 mm of translation on dynamic lumbar radiographs. (2) Presence of degenerative lumbar spine disease findings on MRI, such as ligamentum flavum hypertrophy, facet joint hypertrophy, disc degeneration, or central/foraminal stenosis. (3) Patients with persistent neurological symptoms unresponsive to at least 3 months of appropriate conservative treatment. (4) Undergoing UBE-LIF or conventional open TLIF performed by a senior spinal surgeon. (5) A minimum follow-up of 12 months. The exclusion criteria were as follows: (1) Multi-level lumbar instability. (2) Patients with sagittal imbalance (PI–LL mismatch > 10° or SVA > 5 cm) or those requiring segmental lordosis correction exceeding 10°. (3) Patients who underwent ALIF or long-segment fusion procedures. (4) Previous history of lumbar spinal surgery. (5) Patients with space-occupying lesions in the spinal canal or lumbar tumors. (6) Patients with lumbar infections (including tuberculosis). (7) Follow-up duration of less than 12 months. (8) Patients for whom clinical scores or radiological data were unavailable in hospital records.

### Surgical technique

All surgical procedures were performed in a tertiary care university hospital by the same spine surgeon, experienced in both endoscopic and conventional open surgery. Between January 2021 and May 2024, both UBE-LIF and conventional open TLIF were performed during the same period in our institution. The choice of surgical technique was mainly influenced by logistical factors, such as instrument availability and sterilization status, rather than patient characteristics or surgical complexity. Specifically, UBE-LIF was performed when the percutaneous transpedicular screw fixation set was available; when the required technical equipment was unavailable, conventional open TLIF was performed. All patients received either general or spinal anesthesia. Both types of surgical procedures were performed in the prone position. Prophylactic antibiotics were administered preoperatively.

#### Unilateral biportal endoscopic lumbar interbody fusion (UBE-LIF)

**Fig. 1:**
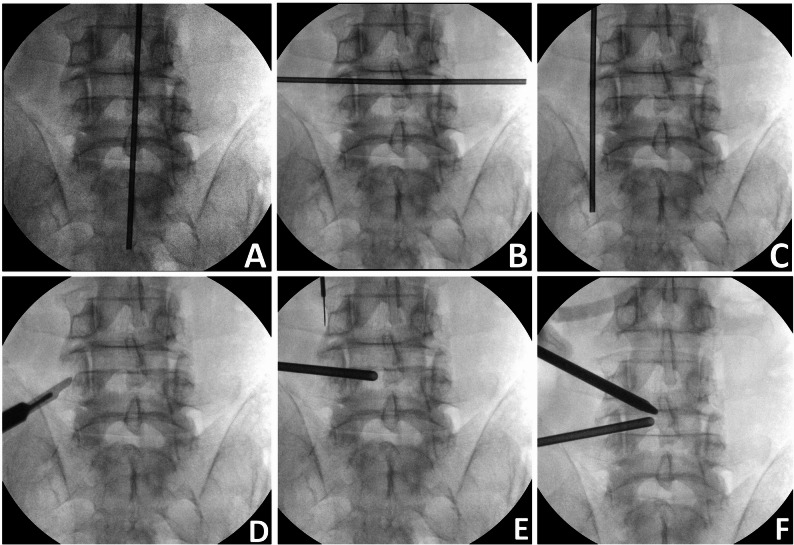
Marking of reference lines in the UBE-LIF technique with the aid of intraoperative fluoroscopy **A** The midline connecting the spinous processes is identified. **B** An anteroposterior fluoroscopic image is obtained by adjusting the lumbar lordosis angle, ensuring that the inferior endplate of the superior vertebra is visualized as a single distinct line, and the target intervertebral level is marked. **C** The line connecting the lateral borders of the ipsilateral pedicles is marked. **D** The working (caudal) portal is created at the level of the inferior vertebral pedicle (~ 10 mm). **E** The viewing (cranial) portal is created at the level of the superior vertebral pedicle (~ 5 mm). **F** The positions of the portals are confirmed using C-arm fluoroscopy

**Fig. 2:**
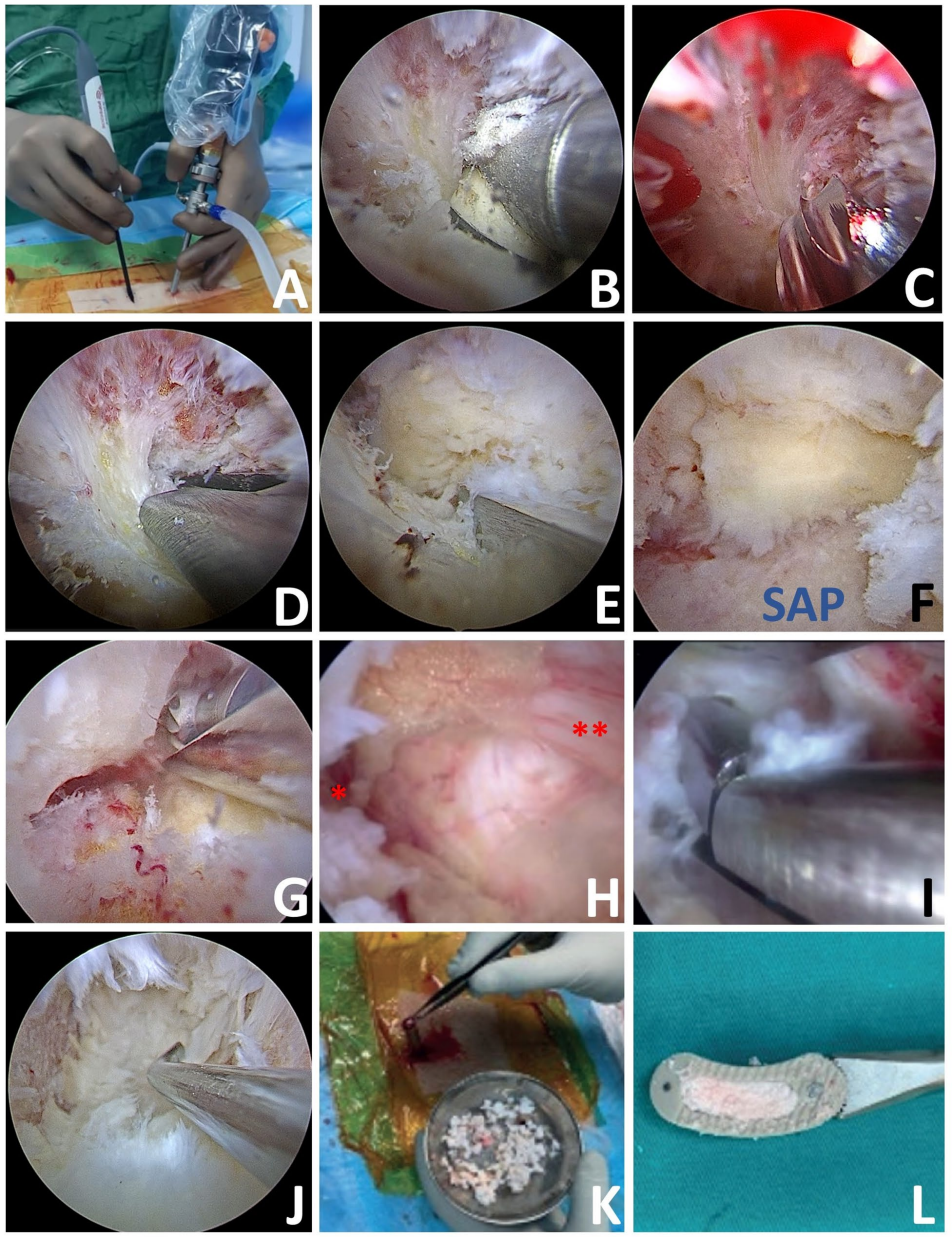
Intraoperative photographs and endoscopic images of the UBE-LIF technique **A** The endoscope is advanced through the cranial portal, and the instruments are advanced through the caudal portal to achieve triangulation on the lamina. **B** The tissues on the lamina are removed using RF ablation. **C** Laminotomy is initiated with a high-speed burr. **D** Laminotomy is continued using a Kerrison rongeur, and autograft is harvested for insertion into the intervertebral space. **E** The inferior articular process is resected using an osteotome and preserved for use as a graft. **F** Intraoperative image after resection of the inferior articular process, showing the superior articular process (SAP) and the ligamentum flavum. The ligamentum flavum is preserved until bone removal is completed to prevent nerve injury. **G** If bilateral decompression is required, contralateral sublaminar laminoplasty is performed while preserving the ligamentum flavum. **H** Endoscopic image showing the disc and nerve roots following removal of the ligamentum flavum. Red asterisks indicate the exiting and traversing nerve roots (*: L4, **: L5). **I** Annulotomy and discectomy are performed after the traversing root is retracted using a root retractor. **J** Endplate preparation is carried out by curette or dissector to remove the cartilaginous surfaces of both vertebral endplates. **K** The prepared grafts are inserted into the intervertebral space with the aid of a 6-mm diameter tube. **L** After selecting a cage of appropriate size, the cage is filled with grafts

**Fig. 3:**
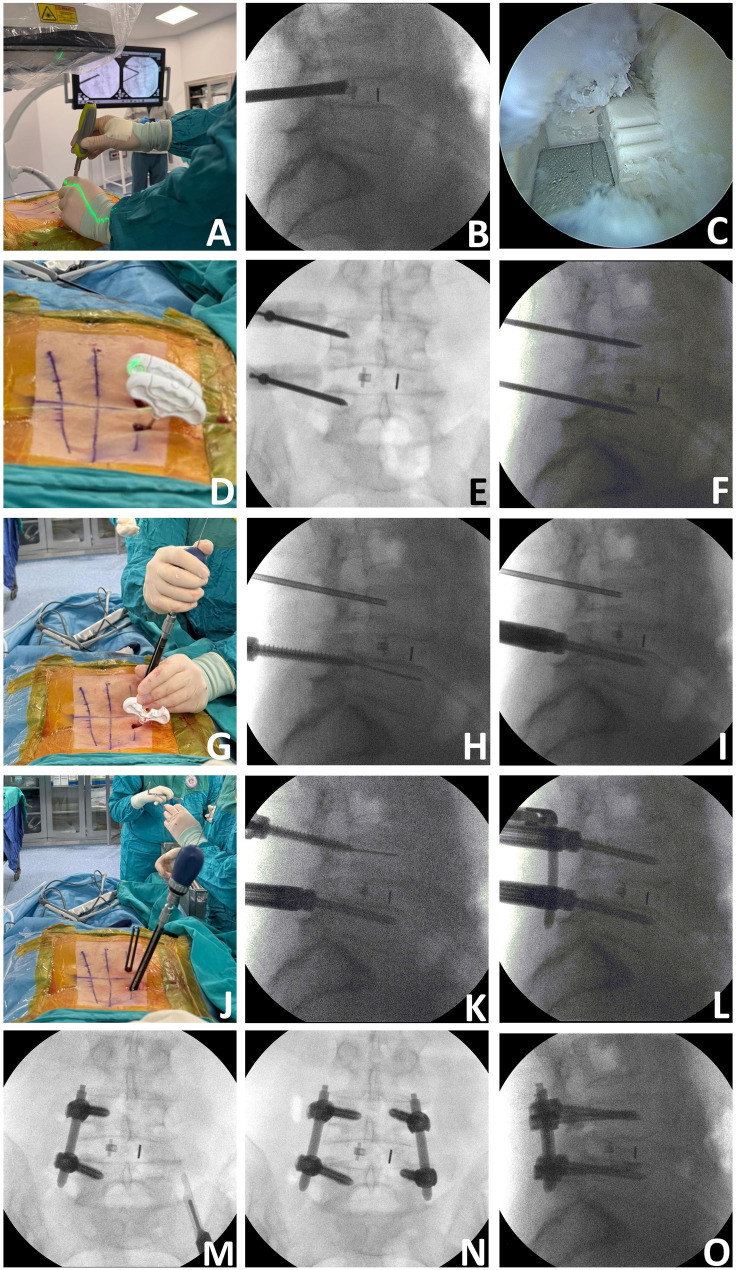
Intraoperative fluoroscopic images of cage placement and percutaneous transpedicular screw fixation in the UBE-LIF technique **A**–**C** Under endoscopic and fluoroscopic guidance, the cage is inserted through the intervertebral space until its posterior border reaches the posterior vertebral body margin, and is then repositioned transversely with the aid of an impactor. **D** After endoscopic decompression and cage placement, Jamshidi needles are inserted into the ipsilateral pedicles through the viewing and working portal incisions. **E**,**F** The correct placement of the Jamshidi needles is confirmed using AP and lateral fluoroscopic images. **G**–**I** A cannulated pedicle screw with an extension sleeve is inserted percutaneously over the guide wire. **J**,**K** The same procedure is repeated for the superior vertebra under fluoroscopic guidance. **L** Using a rod inserter, an appropriately sized rod is inserted percutaneously. **M** Under fluoroscopic guidance, small stab incisions are made on the contralateral side, lateral to the vertical interpedicular line. **N**, **O** Percutaneous transpedicular screw fixation is performed on the contralateral side following the same steps as on the ipsilateral side. Reduction and stabilization are achieved by tightening the screw caps

The preoperatively determined surgical level is identified under fluoroscopic guidance, and reference lines and incision sites are marked, as shown in Fig. 1. Portals are created through two skin incisions of approximately 0.5–1 cm, using a series of dilators. A 0° or 30°, 4.0-mm endoscope (Karl Storz, Tuttlingen, Germany) is introduced through the cranial (viewing) portal, while instruments including an RF ablation probe, burr, and Kerrison punches are inserted via the caudal (working) portal. The surgical steps are detailed in Figs. 2 and 3. Laminotomy is initiated with a high-speed burr and completed with a Kerrison rongeur, allowing autograft harvesting. The inferior articular process is resected with an osteotome and preserved as graft, while only a small medial and superior portion of the superior articular process is removed. The ligamentum flavum is then excised, exposing the exiting and traversing nerve roots. Following retraction of the traversing root with a root retractor, annulotomy and discectomy are performed. The cartilaginous endplate is excised with a curette or dissector, and the prepared grafts are inserted into the anterior disc space. An intervertebral polyetheretherketone (PEEK) fusion cage (Normmed Medical Inc., Ankara, Turkey) of appropriate size is selected, filled with grafts, and inserted into the intervertebral space. The cage is repositioned transversely with the aid of an impactor to achieve a stable and anterior placement within the intervertebral disc space while maintaining midline positioning. After endoscopic decompression and cage placement, cannulated pedicle screws with extension sleeves are inserted percutaneously (Fig. 3). Bilateral percutaneous transpedicular screw fixation (Thoracolumbar MIS Screw System, Normmed Medical Inc., Ankara, Turkey) is then performed under fluoroscopic guidance, according to the standard technique described by Foley and Gupta [26].

#### Conventional open TLIF

The preoperatively planned surgical level is confirmed under fluoroscopic guidance, and a standard midline incision is performed. The TLIF procedure is performed according to the steps described by Harms and Jeszenszky in 1998 [10, 11]. The muscle fascia is incised in alignment with the skin incision, and the paravertebral muscles are carefully retracted to expose the bilateral facet joints and transverse processes. Multiaxial pedicle screws are carefully inserted into the designated anatomical levels on both sides, and proper placement is confirmed with fluoroscopy. A unilateral laminotomy is performed at the intended fusion level. An inferior facetectomy is carried out on the same side. The proximal portion of the superior facet is resected to widen the exposure. The resected fragments are used as autograft, and if necessary, an iliac crest graft can be harvested through the same incision using a small window. In the setting of spinal stenosis, decompression is obtained by removal of the ligamentum flavum. Annulotomy and discectomy are carried out with care to avoid injury to the traversing and exiting nerve roots. The cartilaginous endplates are carefully removed. After distraction of the disc space, the graft material is packed anteriorly, and an intervertebral fusion cage of appropriate size (TLIF PEEK cage, Normmed Medical Inc., Ankara, Turkey) filled with autograft is inserted transforaminally. Compression and fixation are secured with the help of rods. Decortication is carried out on the side contralateral to the hemilaminectomy to facilitate posterior fusion, and additional autografts are placed dorsolaterally in this region. A hemovac drain is inserted, and the wound is closed in layers.

#### Postoperative management

Postoperative management protocols were identical in both treatment groups. Postoperative analgesia was provided using acetaminophen (paracetamol) and non-steroidal anti-inflammatory drugs (NSAIDs). Opioid analgesics were administered if non-opioid agents were insufficient. All patients were mobilized on the first postoperative day.

### Clinical and radiological evaluations

Demographic data, including patient age and sex, level of surgery, operative time, length of hospital stay, blood transfusion requirement, perioperative complications, and laboratory markers of muscle injury (hemoglobin, CRP, ESR, WBC, myoglobin), were recorded from the hospital’s digital archive. Clinical improvement was assessed using the Japanese Orthopaedic Association (JOA) score, the Oswestry Disability Index (ODI), and the Visual Analog Scale (VAS). The VAS was applied in three subcategories: low back pain, ipsilateral leg pain, and contralateral leg pain. The side of the laminotomy was defined as ipsilateral, while the opposite side was defined as contralateral. All clinical measurements were obtained at multiple time points: preoperatively, on postoperative day 10, at 6 months postoperatively, and at 12 months postoperatively.

Preoperative and postoperative radiological evaluations included anteroposterior, lateral, and flexion–extension radiographs (dynamic lumbar radiographs), as well as computed tomography (CT) and magnetic resonance imaging (MRI). All radiological measurements were carried out with a DICOM viewer (POP-net webserver, Image ONE Co., Tokyo, Japan). Patients with ≥ 10° of angular motion or ≥ 4 mm of translation on dynamic lumbar radiographs were considered to have unstable lumbar spondylolisthesis. The status of intervertebral fusion was radiologically evaluated using CT scans at the 12-month postoperative follow-up. The Bridwell grading system was used for this assessment [27]. Grade I represents a solid fusion characterized by remodeling and visible trabecular continuity. Grade II indicates that the graft remains intact with incomplete remodeling but without radiolucent lines. Grade III describes an intact graft with possible lucency at either the cranial or caudal end. Grade IV reflects a lack of fusion, associated with graft collapse or resorption (Supplementary Fig. 1) [27]. Radiological assessments were performed by two blinded spine surgeons. Interobserver agreement for Bridwell grading was excellent, with a quadratic weighted Cohen’s kappa of 0.91 (95% confidence interval [CI], approximately 0.86–0.95). In cases of disagreement, consensus was achieved by a third blinded spine surgeon.

Lumbar paraspinal muscle degeneration was assessed on preoperative and 12-month postoperative magnetic resonance imaging (MRI) and graded using the Goutallier classification. The Goutallier classification is a visual grading system for qualitative assessment of fatty infiltration. While it was originally developed for the rotator cuff muscles, its use has subsequently been extended to the paraspinal muscles [28, 29]. The grading system was defined as follows: grade 0, no intramuscular fat; grade 1, minimal fatty streaks; grade 2, fat present but less than muscle tissue; grade 3, fat and muscle in equal amounts; and grade 4, fat exceeding the amount of muscle tissue [28, 29]. For this classification, T1-weighted axial MRI images at the surgically treated intervertebral disc level were used for Goutallier grading on both preoperative and postoperative MRI examinations. The muscle composition of the bilateral paraspinal muscles, including the multifidus and erector spinae, was independently assessed by two blinded spine surgeons and classified into five grades based on the visually evaluated fat-to-muscle ratio at the operative intervertebral disc level. Interobserver agreement for Goutallier grading was excellent, with a quadratic weighted Cohen’s kappa of 0.88 (95% confidence interval [CI], 0.82–0.92). In the event of disagreement, consensus was reached by a third blinded spine surgeon.

### Statistical analysis

Statistical analysis was performed using SPSS v.22.0 (SPSS Inc., Chicago, IL). Descriptive statistics were used to summarize the demographic characteristics of the patients, with means and standard deviations reported for continuous variables. The Shapiro–Wilk test was applied to assess normality. For numerical data, between-group comparisons were performed using Student’s t-test, whereas within-group changes over time were analyzed with repeated measures ANOVA. For categorical data, the Chi-square test or Fisher’s exact test was applied as appropriate. A *p*-value of less than 0.05 was considered statistically significant.

## Results

A total of 100 patients were evaluated in this study. The patients consisted of 28 males (28.0%) and 72 females (72.0%) with a mean age of 62.8 years. Among the patients who met the inclusion criteria, 26 underwent conventional open TLIF, while 74 underwent UBE-LIF. Table 1 presents the patient characteristics and demographics of the two treatment groups. The minimum follow-up period was 12 months. No significant differences were observed in age, sex, lesion level, and mean follow-up duration (*p* > 0.05; Table 1). The mean operative time was 195.4 min in the conventional open TLIF group and 154.2 min in the UBE-LIF group (*p* = 0.005). The mean operative time was 41.2 min shorter in the UBE-LIF group than in the conventional open TLIF group (95% CI: 13.9–68.5, *p* = 0.005). In the UBE-LIF group, no patient required postoperative blood transfusion, while blood transfusion was required in 3 patients (11.5%) in the conventional open TLIF group (*p* = 0.003). Length of hospital stay was significantly lower in the UBE-LIF group compared with the conventional open TLIF group (45.4 h vs. 98.6 h, *p* < 0.001). The mean length of hospital stay was 53.2 h shorter in the UBE-LIF group than in the open TLIF group (95% CI 35.6–70.8, *p* < 0.001). Postoperative opioid analgesics were required in 20 patients (76.9%) in the conventional open TLIF group, whereas only 11 patients (14.8%) in the UBE-LIF group required opioid therapy. Postoperative opioid analgesics were required significantly less frequently in the UBE-LIF group than in the conventional open TLIF group, with an absolute risk reduction of 62.1% (95% CI 43.9–80.1, *p* < 0.001).

**Table 1:** Demographics and patient characteristics

		Conventional Open TLIF (n = 26)	UBE-LIF (n = 74)	*p* value
Age		60.5 (± 12.9)	63.6 (± 11.7)	0.275
Sex	Male	7 (26.9%)	21 (28.4%)	0.876
	Female	19 (73.1%)	53 (71.6%)	
Level	L1-2	1 (3.8%)	2 (2.7%)	0.460
	L2-3	2 (7.7%)	4 (5.4%)	
	L3-4	3 (11.5%)	18 (24.3%)	
	L4-5	11 (42.3%)	47 (63.5%)	
	L5-S1	9 (34.6%)	3 (4.1%)	
Operative time (minutes)		195.4 (± 59.2)	154.2 (± 66.4)	**0.005**
Blood transfusion	Yes	3 (11.5%)	0	**0.003**
	No	23 (88.5%)	74 (100%)	
Length of hospital stay (hours)		98.6 (± 42.5)	45.4 (± 23.6)	** < 0.001**
Follow up (months)		26.5 (± 8.1)	24.4 (± 6.2)	0.237
Complication	Yes	1 (3.8%)	2 (2.7%)	0.769
	No	25 (96.2%)	72 (97.3%)	

Student’s *t*-test was used for the comparison of numerical data. The chi-square test or Fisher’s exact test was used for categorical data. A *p*-value < 0.05 was considered statistically significant. Statistically significant *p*-values (< 0.05) are indicated in bold

*UBE-LIF* Unilateral biportal endoscopic lumbar interbody fusion; *TLIF* Transforaminal lumbar interbody fusion

The comparison of clinical scores between the treatment groups is presented in Table 2. There were no statistically significant differences between the two groups in VAS, JOA, or ODI scores at baseline, on postoperative day 10, at 6 months postoperatively, and at 12 months postoperatively (*p* > 0.05; Table 2). Both groups showed statistically significant improvements in low back pain VAS, leg pain VAS, ODI, and JOA scores postoperatively (*p* < 0.001; Table 2). According to a review of the existing literature, the minimum clinically important difference (MCID) thresholds were determined to be 1.6 for VAS back pain, 1.7 for VAS leg pain, and 14.3 for ODI [30]. Our findings on postoperative improvement in clinical scores met the MCID thresholds in both treatment groups (Table 2).

**Table 2:** Comparison of clinical scores between the treatment groups

		Conventional Open TLIF (n = 26)	UBE-LIF (n = 74)	*p* value*
Low back pain VAS				
	Preop	6.15 (± 1.82)	6.41 (± 1.72)	0.529
	Postop. 10th day	4.46 (± 1.55)	4.27 (± 1.49)	0.590
	Postop. 6th month	2.04 (± 1.80)	1.81 (± 1.09)	0.544
	Postop. 12th month	1.83 (± 1.47)	1.68 (± 1.24)	0.611
	*p* value**	** < 0.001**	** < 0.001**	
Ipsilateral leg pain VAS				
	Preop	4.96 (± 2.80)	5.00 (± 2.30)	0.948
	Postop. 10th day	3.27 (± 2.34)	2.97 (± 2.06)	0.566
	Postop. 6th month	1.88 (± 1.75)	1.80 (± 1.28)	0.832
	Postop. 12th month	1.79 (± 1.58)	1.72 (± 1.43)	0.846
	*p* value**	** < 0.001**	** < 0.001**	
Contralateral leg pain VAS				
	Preop	4.12 (± 2.56)	5.00 (± 2.64)	0.142
	Postop. 10th day	2.12 (± 1.68)	2.24 (± 2.10)	0.771
	Postop. 6th month	1.77 (± 1.45)	2.07 (± 1.31)	0.358
	Postop. 12th month	1.83 (± 1.62)	1.98 (± 1.57)	0.227
	*p* value**	** < 0.001**	** < 0.001**	
JOA Score				
	Preop	10.58 (± 2.95)	9.27 (± 2.62)	0.052
	Postop. 10th day	14.69 (± 4.13)	14.16 (± 4.05)	0.575
	Postop. 6th month	17.00 (± 3.76)	16.64 (± 4.42)	0.690
	Postop. 12th month	17.82 (± 4.08)	17.38 (± 3.93)	0.740
	*p* value**	** < 0.001**	** < 0.001**	
Oswestry disability index (ODI)				
	Preop	63.85 (± 16.75)	64.86 (± 13.67)	0.783
	Postop. 10th day	40.77 (± 11.63)	41.62 (± 14.04)	0.763
	Postop. 6th month	25.38 (± 14.20)	26.22 (± 15.76)	0.802
	Postop. 12th month	24.13 (± 14.20)	24.87 (± 15.76)	0.794
	*p* value**	** < 0.001**	** < 0.001**	

Between-group comparisons were performed using Student’s *t*-test, whereas within-group changes over time were analyzed with repeated measures ANOVA. p* values indicate between-group comparisons, while p** values denote within-group comparisons. Statistically significant *p*-values (< 0.05) are indicated in bold

*UBE-LIF* Unilateral biportal endoscopic lumbar interbody fusion; *TLIF* Transforaminal lumbar interbody fusion; *VAS* Visual analog scale; *JOA* Japanese Orthopaedic Association; *ODI* Oswestry disability index

The comparison of laboratory parameters between the treatment groups is presented in Table 3. Preoperatively, there were no statistically significant differences between the two groups in hemoglobin, WBC, CRP, ESR, and myoglobin values (*p* > 0.05; Table 3). The mean hemoglobin value on postoperative day 0 was significantly lower in the conventional open TLIF group compared with the UBE-LIF group (11.35 vs. 12.23 g/dL, *p* = 0.024). Mean hemoglobin levels on postoperative day 0 were 0.88 g/dL lower in the open TLIF group than in the UBE-LIF group (95% CI: 0.14–1.62, *p* = 0.024). Similarly, on postoperative day 10, the mean hemoglobin level was significantly lower in the conventional open TLIF group compared with the UBE-LIF group (10.75 vs. 12.65, *p* < 0.001). Mean hemoglobin levels on postoperative day 10 were 1.90 g/dL lower in the open TLIF group than in the UBE-LIF group (95% CI: 1.18–2.62, *p* < 0.001). On postoperative day 0, a significantly higher mean WBC was observed in the conventional open TLIF group compared with the UBE-LIF group (13.39 vs. 10.95 × 10^3^/µL, p = 0.003). Mean WBC levels on postoperative day 0 were 2.44 × 10^3^/µL higher in the open TLIF group than in the UBE-LIF group (95% CI 0.92–3.96, *p* = 0.003). Likewise, on postoperative day 0, the mean myoglobin level was significantly higher in the conventional open TLIF group compared with the UBE-LIF group (454.13 vs. 281.48 ng/mL, *p* = 0.005). Mean myoglobin levels on postoperative day 0 were 172.7 ng/mL higher in the open TLIF group than in the UBE-LIF group (95% CI: 65.3–280.1, *p* = 0.005). Furthermore, on postoperative day 10, CRP and ESR values were significantly lower in the UBE-LIF group compared with the conventional open TLIF group (*p* = 0.008 and *p* = 0.012, respectively; Table 3). Mean CRP levels on postoperative day 10 were 19.9 mg/L higher in the open TLIF group than in the UBE-LIF group (95% CI: 6.2–33.6, *p* = 0.008). Similarly, mean ESR levels on postoperative day 10 were 8.47 mm/h higher in the open TLIF group than in the UBE-LIF group (95% CI 1.2–15.7, *p* = 0.012).

**Table 3:** Comparison of laboratory parameters between the treatment groups

		Conventional Open TLIF (n = 26)	UBE-LIF (n = 74)	*p* Value*
Hemoglobin (g/dL)				
	Preop	13.59 ± 1.50	13.68 ± 1.51	0.770
	Postop. day 0	11.35 ± 1.60	12.23 ± 1.79	**0.024**
	Postop. 10th day	10.75 ± 1.55	12.65 ± 1.80	** < 0.001**
	*p* value**	** < 0.001**	** < 0.001**	
WBC (10^3^/µL)				
	Preop	7,86 ± 2,20	7,14 ± 1,85	0.146
	Postop. day 0	13,39 ± 3,56	10,95 ± 3,01	**0.003**
	Postop. 10th day	9,10 ± 1,96	8,37 ± 2,15	0.115
	*p* value**	** < 0.001**	** < 0.001**	
CRP (mg/L)				
	Preop	4.63 ± 7.71	4.01 ± 7.21	0.703
	Postop. day 0	24.97 ± 27.31	13.61 ± 21.75	0.060
	Postop. 10th day	28.61 ± 34.09	8.72 ± 17.75	**0.008**
	*p* value**	** < 0.001**	** < 0.001**	
Erythrocyte sedimentation rate (ESR) (mm/h)				
	Preop	14.18 ± 13.75	12.65 ± 14.99	0.614
	Postop. day 0	23.31 ± 17.54	17.39 ± 14.34	0.130
	Postop. 10th day	25.46 ± 13.03	16.99 ± 17.67	**0.012**
	*p* value**	** < 0.001**	**0.003**	
Myoglobin (ng/mL)				
	Preop	60.50 ± 100.73	70.30 ± 106.42	0.667
	Postop. day 0	454.13 ± 263.00	281.48 ± 219.61	**0.005**
	Postop. 10th day	154.92 ± 230.78	92.02 ± 117.47	0.187
	*p* value**	**0.007**	**0.002**	

Between-group comparisons were performed using Student’s *t*-test, whereas within-group changes over time were analyzed with repeated measures ANOVA. p* values indicate between-group comparisons, while p** values denote within-group comparisons. Statistically significant *p*-values (< 0.05) are indicated in bold

*UBE-LIF* Unilateral biportal endoscopic lumbar interbody fusion; *TLIF* Transforaminal lumbar interbody fusion; *WBC* White blood cell; *CRP* C-reactive protein; *ESR* Erythrocyte sedimentation rate

The status of intervertebral fusion was evaluated using computed tomography (CT) scans obtained at the 12-month follow-up. According to the Bridwell grading system, the conventional open TLIF group consisted of 13 (50.0%), 11 (42.3%), 2 (7.7%), and 0 cases of Grades I, II, III, and IV, respectively, whereas the UBE-LIF group comprised 44 (59.5%), 30 (40.5%), 0, and 0 cases of Grades I, II, III, and IV, respectively (Table 4). Grades I–II were considered satisfactory fusion, while Grades III–IV were considered fusion failure. No statistically significant difference in fusion rates was found between the UBE-LIF and conventional open TLIF groups (100% vs 92.3%, respectively; *p* = 0.066).

**Table 4:** Comparison of postoperative 12th-month fusion status between the treatment groups

		Conventional Open TLIF (n = 26)	UBE-LIF (n = 74)	*p* value
Fusion (n,%)		24 (92.3%)	74 (100%)	0.066
Bridwell Fusion Grade	Grade I	13 (50.0%)	44 (59.5%)	0.066
	Grade II	11 (42.3%)	30 (40.5%)	
	Grade III	2 (7.7%)	0 (0%)	
	Grade IV	0 (0%)	0 (0%)	

Intervertebral fusion status was evaluated radiologically at the 12-month postoperative follow-up using CT scans and classified according to the Bridwell grading system. Grades I–II were considered satisfactory fusion, whereas Grades III–IV were considered a failure

Fisher’s exact test was used for the comparison of categorical data. A *p*-value > 0.05 was considered not statistically significant

*UBE-LIF* Unilateral biportal endoscopic lumbar interbody fusion; *TLIF* Transforaminal lumbar interbody fusion

The preoperative and postoperative status of lumbar paraspinal muscle degeneration in both groups is presented in Table 5. Lumbar paraspinal muscle degeneration was assessed on magnetic resonance imaging (MRI) and graded using the Goutallier classification. Preoperative evaluation showed no significant difference in the distribution of grades between the groups (*p* = 0.552; Table 5). At the 12-month postoperative MRI, grade distribution (Grades 0–IV) in the conventional TLIF group was 2 (7.7%), 5 (19.2%), 13 (50.0%), 5 (19.2%), and 1 (3.8%), whereas in the UBE-LIF group it was 46 (62.1%), 15 (20.3%), 13 (17.6%), 0, and 0, respectively (*p* < 0.001; Table 5). Postoperative progression of Goutallier grade was not observed in the UBE-LIF group, whereas it occurred in 21 patients (80.8%) in the conventional open TLIF group (*p* < 0.001). This corresponded to an absolute risk difference of 80.8% (95% CI: 65.6–96.0).

**Table 5:** Comparison of preoperative and postoperative lumbar paraspinal muscle degeneration between the treatment groups

		Conventional Open TLIF (n = 26)	UBE-LIF (n = 74)	*p* value*
Preop. Goutallier Grade*	Grade 0	20 (76.9%)	46 (62.1%)	0.552
	Grade 1	3 (11.5%)	15 (20.3%)	
	Grade 2	3 (11.5%)	13 (17.6%)	
	Grade 3	0 (0%)	0 (0%)	
	Grade 4	0 (0%)	0 (0%)	
Postop. Goutallier Grade*	Grade 0	2 (7.7%)	46 (62.1%)	** < 0.001**
	Grade 1	5 (19.2%)	15 (20.3%)	
	Grade 2	13 (50.0%)	13 (17.6%)	
	Grade 3	5 (19.2%)	0 (0%)	
	Grade 4	1 (3.8%)	0 (0%)	
*p* value**	** < 0.001**	<0.001		

Lumbar paraspinal muscle degeneration was evaluated preoperatively and at the 12-month postoperative follow-up using MRI and classified according to the Goutallier classification system

As more than 20% of the expected counts were below 5, the chi-square assumptions were not met; thus, categories were combined into low grade (Grade 0–I) and high grade (Grade II–IV), and Fisher’s exact test was used. p* values indicate between-group comparisons, while p** values denote within-group comparisons. Statistically significant *p*-values (< 0.05) are indicated in bold

*UBE-LIF* Unilateral biportal endoscopic lumbar interbody fusion; *TLIF* Transforaminal lumbar interbody fusion

In the UBE-LIF group, there was one case of dural tear and one case of epidural hematoma, with no postoperative infections observed. In the conventional open TLIF group, there was one case of superficial surgical site infection. Overall, there was no statistically significant difference in the incidence of complications between the two groups (2.7% vs. 3.8%, *p* = 0.769). In the patient who developed an intraoperative dural tear during UBE-LIF, endoscopic dural repair was performed without the need for conversion to open surgery. No patient experiencing complications required additional surgery in either group.

## Discussion

Interbody fusion is the standard surgical treatment for degenerative lumbar instability [2, 3, 6]. Various surgical techniques for lumbar interbody fusion have been developed over the years, each offering unique advantages and limitations [3, 5, 7–9]. Posterior lumbar interbody fusion (PLIF) was first described by Cloward in the 1950s and became widely used in spinal surgery owing to its satisfactory outcomes [2, 31]. Although PLIF achieved satisfactory fusion outcomes, its requirement for bilateral laminectomy and extensive tissue dissection led to the pursuit of less invasive techniques [2, 8]. TLIF was developed as a unilateral modification of PLIF and was popularized by Harms and Jeszenszky as an alternative to PLIF [10, 11, 13]. By utilizing a unilateral transforaminal approach, TLIF minimizes retraction of the dural sac and nerve roots, thereby reducing the risk of root-related complications [2, 8, 10–14]. Owing to its advantages, TLIF has gained widespread use and is considered a standard surgical technique for lumbar interbody fusion [1, 32].

Minimally invasive techniques were developed to overcome the drawbacks of conventional open procedures, including iatrogenic paraspinal muscle damage, significant blood loss, and prolonged recovery due to extensive tissue dissection [1, 2, 5, 6, 19]. The tubular retractor system was developed by Foley and Smith in 1994 and was initially applied to discectomy. This approach enabled discectomy to be performed without a traditional midline incision, thereby preserving the midline musculoligamentous supporting structures [15, 33]. In 2002, Foley and Gupta described the technique of percutaneous transpedicular screw fixation [26]. These technical advances facilitated the minimally invasive application of TLIF without the need for conventional open approaches, and in 2003, Foley et al. introduced the minimally invasive transforaminal lumbar interbody fusion (MIS-TLIF) technique [15]. MIS-TLIF is carried out through a small paramedian incision with the aid of a tubular retractor [2, 3, 15]. Foley et al. stated that visualization could be achieved with a microscope, loupes, or a microendoscope, depending on the surgeon’s preference [15]. Despite its advantages, MIS-TLIF is challenged by the limited working space and narrow field of view through the tubular retractor, particularly in deep operative fields [2, 3].

In recent years, advances in optical technologies have led to the rapid development and widespread use of endoscopic spinal surgery techniques [20–23]. Among these, unilateral biportal endoscopy (UBE) has emerged as a safe and effective method, offering distinct advantages [1–3, 5, 6, 20–23]. In a series of 215 patients with spinal stenosis without instability, Merter et al. comprehensively compared the three most commonly used endoscopic techniques, including UBE, microendoscopy, and uniportal percutaneous endoscopy, both radiologically and clinically [21]. They reported that although all three techniques provided effective decompression, UBE proved to be the most effective for contralateral lateral recess decompression [21]. Separate working and viewing portals in UBE provide a geometric advantage and facilitate the independent movement of surgical instruments [21, 24]. UBE also offers high flexibility in instrument maneuverability, as it does not require a tubular retractor like microendoscopy [20, 21]. In addition, since it does not involve a small-diameter outer cannula and working channel as in uniportal percutaneous endoscopy, larger instruments such as Kerrison punches can be used. A key advantage of UBE is that it can be performed using a standard 4.0-mm rigid endoscope, commonly employed in knee and shoulder arthroscopy, without requiring specialized endoscopic instruments or optics [21, 24]. Owing to these advantages, UBE has gained widespread use and has been effectively applied in lumbar spine disorders such as intervertebral disc herniation and decompression of the spinal canal or foraminal stenosis [20–23]. More recently, it has been introduced for lumbar interbody fusion under the term UBE-LIF [1–3, 5, 6].

The UBE-LIF technique was first introduced and reported with preliminary clinical results by Heo et al. in 2017 [24]. They reported that in a case series of 69 patients who underwent single-level fusion with a mean follow-up of 13.5 months, adequate neural decompression was achieved with significant improvements in VAS and ODI scores, without postoperative neurological deterioration [24]. Since then, numerous studies in the literature have reported that UBE-LIF is a safe and effective minimally invasive technique for degenerative lumbar spine disease [1–3, 5, 6, 19, 24, 25]. However, only a limited number of studies have compared UBE-LIF with conventional open TLIF, both radiologically and clinically [3, 6].

In the study by Zheng et al. UBE-LIF was performed in 58 patients and open TLIF in 70 patients. They reported postoperative improvement in VAS and ODI scores in both groups, with no significant difference in fusion rates (98.27% vs. 98.57%) [6]. They also stated reduced blood loss and shorter hospital stay in the UBE-LIF group. In a similar study, Lu et al. examined the outcomes of 79 patients with lumbar spondylolisthesis who underwent UBE-LIF, MIS-TLIF, or open TLIF. They reported that all three groups showed significant postoperative improvement in clinical scores [3]. No significant difference was found in postoperative fusion rates among the UBE-LIF, MIS-TLIF, and open TLIF groups, which were reported as 88.5%, 89.2%, and 92.0%, respectively [3]. They also mentioned that postoperative blood loss from the hemovac drain was lower in the UBE-LIF group [3]. Consistent with the literature, our study revealed significant postoperative improvement in clinical scores in both the UBE-LIF and conventional open TLIF groups, with similar fusion rates observed in both (100% and 92.3%, respectively). The relatively high fusion rate observed in the UBE-LIF group in our series may be related to several factors. First, fusion assessment was performed at the 12-month postoperative follow-up, and studies with longer follow-up durations may demonstrate different distributions of fusion grades (e.g., some fusions initially appearing as Grade II may later be reclassified as Grade III due to the development of radiolucency over time). Therefore, the follow-up duration limited to 12 months may have contributed to the higher observed fusion rate. Second, the endoscopic nature of the UBE-LIF technique allows enhanced visualization of the intervertebral disc space, which may facilitate more effective preparation of the vertebral endplates compared with conventional open techniques. Direct visualization during endoscopic disc space preparation may allow more thorough removal of the cartilaginous surfaces of the vertebral endplates, potentially creating more favorable conditions for intervertebral fusion. In addition, proper cage size selection and optimal positioning within the disc space are important factors for achieving successful fusion. With sufficient endoscopic experience, the UBE-LIF technique does not represent a disadvantage in this regard. Finally, all procedures in our series were performed by the same senior spine surgeon experienced in both endoscopic and conventional open spinal surgery, which may also have contributed to the relatively high fusion rate observed in our cohort.

In line with prior studies, our findings demonstrated that intraoperative blood loss and hospital stay were more favorable in the UBE-LIF group. UBE-LIF is a minimally invasive procedure that utilizes small incisions and causes less iatrogenic paraspinal muscle damage. Accordingly, lower intraoperative blood loss compared with open TLIF is an expected finding. In our study, preoperative hemoglobin levels were similar between the groups; however, on postoperative day 0, levels were significantly lower in the open TLIF group. By postoperative day 10, hemoglobin in the UBE-LIF group showed a trend toward recovery, whereas it remained low in the open TLIF group. These findings may be attributable to iatrogenic muscle injury and hidden intramuscular bleeding associated with the open surgery group. In our study cohort, erythrocyte suspension transfusion was administered when postoperative hemoglobin fell below 8 g/dL or when hemodynamic deterioration (tachycardia, hypotension) occurred. Although all patients experienced a postoperative decrease in hemoglobin, transfusion was required in only three cases, all of which were in the conventional open TLIF group.

CRP and ESR are key laboratory indicators of the acute-phase inflammatory response [34–36]. Numerous studies have investigated postoperative changes in CRP and ESR in spinal surgery patients without postoperative infection. Multiple studies have shown that more invasive spinal surgeries result in higher postoperative CRP levels [34, 37]. It is well established that CRP and ESR levels rise rapidly after surgery and gradually decline thereafter. Most studies report that CRP and ESR reach their peak within 3–5 days after surgery. CRP levels gradually decline, returning to normal by the fourth postoperative week, while ESR levels decrease more slowly, normalizing around the sixth postoperative week [34–37]. In contrast, serum myoglobin, a marker of skeletal muscle injury, typically peaks on the first postoperative day, reflecting acute perioperative muscle damage, and then gradually decreases [38]. Consistent with the literature, our study group also showed postoperative increases in CRP, ESR, and myoglobin levels. On postoperative day 0, the mean myoglobin level was significantly higher in the conventional open TLIF group. Furthermore, on postoperative day 10, CRP and ESR values were significantly lower in the UBE-LIF group compared with the conventional open TLIF group. These findings may be explained by reduced postoperative iatrogenic muscle damage in UBE-LIF. Blood tests were obtained when patients presented to the outpatient clinic on the 10th postoperative day for wound evaluation. Therefore, in addition to preoperative and first postoperative day values, we only have blood test results from the 10th postoperative day. As our study is retrospective, blood test results from the 3rd and 5th postoperative days are not available for patients who were discharged earlier. Consequently, data from the period when CRP and ESR likely peaked are not included in our study. A prospective study with a larger cohort would provide further clarification on this issue.

Zheng et al. reported that, at 1 week postoperatively, back pain was significantly improved in the UBE-LIF group compared with the TLIF group, whereas back pain VAS, leg pain VAS, and ODI scores showed no significant differences between the groups at later follow-up assessments [6]. Similarly, Lu et al. found that back pain VAS scores at 1 week postoperatively were significantly better in the UBE-LIF group than in the other groups, while no significant differences in clinical scores were observed between the groups at subsequent follow-up visits [3]. In our study, the low back pain VAS score on postoperative day 10 was lower in the UBE-LIF group than in the open TLIF group (4.27 vs. 4.46), although the difference was not statistically significant. Opioid analgesic requirements were higher in the open TLIF group than in the UBE-LIF group during the early postoperative period. The higher need for opioid analgesia in the open TLIF group suggests that pain intensity was indeed greater in this group during the immediate postoperative period, which aligns with previous studies reporting better early postoperative pain outcomes with UBE-LIF. However, the greater use of opioids in the open TLIF group may have blunted the expected difference in VAS scores by day 10. Consistent with other studies, our study also found no significant differences between the treatment groups in clinical scores such as leg pain VAS, ODI, and JOA across follow-up periods.

A notable difference between our study and the previous studies was observed in the operative time results. Both Zheng et al. [6] and Lu et al. [3] reported that operative times were longer in the UBE-LIF group than in the open TLIF group. For single-level fusion, the mean operative time in the UBE-LIF group was reported as 169.1 min by Zheng et al. and 184.3 min by Lu et al. [3, 6]. In our study, the operative time for the UBE-LIF group was 154.2 min, and, in contrast to previous reports, the operative time in the UBE-LIF group was shorter than that in the open TLIF group. We believe that the surgeon’s experience in endoscopic spine surgery and percutaneous screw fixation plays a critical role in operative time. In our study, all procedures were performed by a senior spine surgeon with extensive experience in both techniques. For surgeons with such expertise, UBE-LIF may offer a time advantage, as it does not require extensive muscle dissection during exposure, as in conventional open TLIF. In addition, continuous irrigation and high-resolution visualization may facilitate the decompression step in UBE compared with the conventional open technique. Moreover, because skin incisions are limited to portal entries, skin closure in UBE-LIF is considerably simpler. In contrast, conventional open TLIF requires multilayer anatomical closure, which may further increase operative time. We consider screw fixation to be the most critical determinant of operative time between the two techniques. For surgeons experienced in percutaneous screw fixation, UBE-LIF may offer a time advantage. However, if the learning curve for percutaneous screw fixation has not yet been completed, operative time may be prolonged. In our study, the surgical team’s extensive experience in percutaneous screw fixation may represent the most important factor contributing to the shorter operative time observed for UBE-LIF.

The evaluation of 12-month postoperative MRI findings to compare Goutallier grades between treatment groups represents a strength of this study. The studies by Zheng et al. [6] and Lu et al. [3] did not report data on Goutallier grades or paraspinal muscle degeneration. In the present study, postoperative paraspinal muscle degeneration was less pronounced in the UBE-LIF group compared with the open TLIF group. It is known that iatrogenic muscle injury and degeneration may contribute to persistent low back pain and poor clinical outcomes following surgery [15, 39]. However, this relationship was not reflected in our 12-month clinical results, as no significant differences were observed between the groups in VAS, ODI, or JOA scores. One possible explanation for this discrepancy is that functional impairment related to paraspinal muscle degeneration may become more evident over longer periods. Postoperative pain and functional recovery are multifactorial and may not be solely determined by paraspinal muscle status during the early recovery phase. Therefore, a 12-month follow-up period may be insufficient to detect the delayed clinical impact of differences in muscle preservation, which may become more apparent in longer-term assessments. Accordingly, while our radiological findings suggest better muscle preservation in the UBE-LIF group, the clinical relevance of this difference remains uncertain within the current follow-up period, and longer-term studies are needed to clarify this relationship.

Our study has several limitations. First, it was a single-center, retrospective study with a relatively small sample size. The imbalance in group sizes and the absence of statistical adjustment methods, such as propensity score matching, may be considered a limitation in adequately addressing potential selection bias. The choice of surgical technique was mainly influenced by logistical factors, such as instrument availability and sterilization status, rather than by patient characteristics or surgical complexity. Baseline demographic and clinical characteristics were comparable between the groups, with no statistically significant differences observed. Therefore, we believe that the impact of selection bias on the main findings is likely to be limited. Nevertheless, the results should be interpreted with caution, and prospective, randomized studies with balanced group allocation are needed to provide more robust evidence. Another limitation of our study is that the findings are restricted to the 12-month postoperative follow-up. Fusion assessment was performed only at this single postoperative time point, and sequential radiological evaluations at earlier and later postoperative intervals were not available. Such staged radiological assessments could provide additional information regarding fusion dynamics and healing progression. In addition, quantitative analysis of cage positioning parameters was not performed, which may represent another limitation when interpreting the radiological outcomes. Additional long-term data are needed to provide a more comprehensive understanding of the clinical outcomes. Furthermore, because of the limited number of patients in the study, particularly at each individual lumbar level, subgroup stratified analyses could not be performed. Finally, the lack of information regarding the learning curve of these techniques constitutes another limitation of our study. All procedures were performed by a senior spine surgeon with extensive experience in endoscopy and percutaneous screw fixation. However, for less experienced surgeons, UBE-LIF may require a learning period, and caution should be exercised when generalizing our findings to this group. Future prospective studies involving larger patient cohorts and extended follow-up periods would offer more comprehensive and reliable insights.

## Conclusion

UBE-LIF and conventional open TLIF provide similar clinical and radiological outcomes in the treatment of degenerative lumbar disease with instability. However, UBE-LIF was associated with less intraoperative blood loss, shorter hospitalization, and less postoperative muscle degeneration. As a minimally invasive technique, UBE-LIF appears to be a strong alternative to conventional open TLIF.

## Supplementary Information

Below is the link to the electronic supplementary material.

Supplementary Fig. 1: Representative examples of fusion grades according to the Bridwell grading system. **A** Grade I: Solid fusion with trabecular continuity and remodeling. **B** Grade II: Intact graft with incomplete remodeling and no radiolucent lines. **C** Grade III: Potential radiolucency at the graft–endplate interface (white arrow). Grade IV (graft collapse or resorption indicating nonunion) was not observed in our series

## Data Availability

The data that support the findings of this study are available from the corresponding author upon reasonable request.

## Abbreviations

ALIF: Anterior lumbar interbody fusion
CI: Confidence interval
CT: Computed tomography
JOA: Japanese Orthopaedic Association
LL: Lumbar lordosis
LLIF: Lateral lumbar interbody fusion
MIS-TLIF: Minimally invasive transforaminal lumbar interbody fusion
MRI: Magnetic resonance imaging
ODI: Oswestry disability index
OLIF: Oblique lumbar interbody fusion
PEEK: Poly ether ether ketone
PI: Pelvic incidence
PLIF: Posterior lumbar interbody fusion
Preop: Preoperative
Postop: Postoperative
RF: Radiofrequency
SVA: Sacral vertical axis
TLIF: Transforaminal lumbar interbody fusion
UBE: Unilateral biportal endoscopy
UBE-LIF: Unilateral biportal endoscopic lumbar interbody fusion
VAS: Visual analog scale
