# Supplementary figures and images for: Comparison of unilateral biportal endoscopic lumbar interbody fusion (UBE-LIF) and conventional open TLIF in degenerative lumbar spine disease: a radiological, clinical, and laboratory study
Source: J Orthop Surg Res. 2026 May 12;21:413. doi: 10.1186/s13018-026-06946-6 (PMC13374138; doi:10.1186/s13018-026-06946-6)

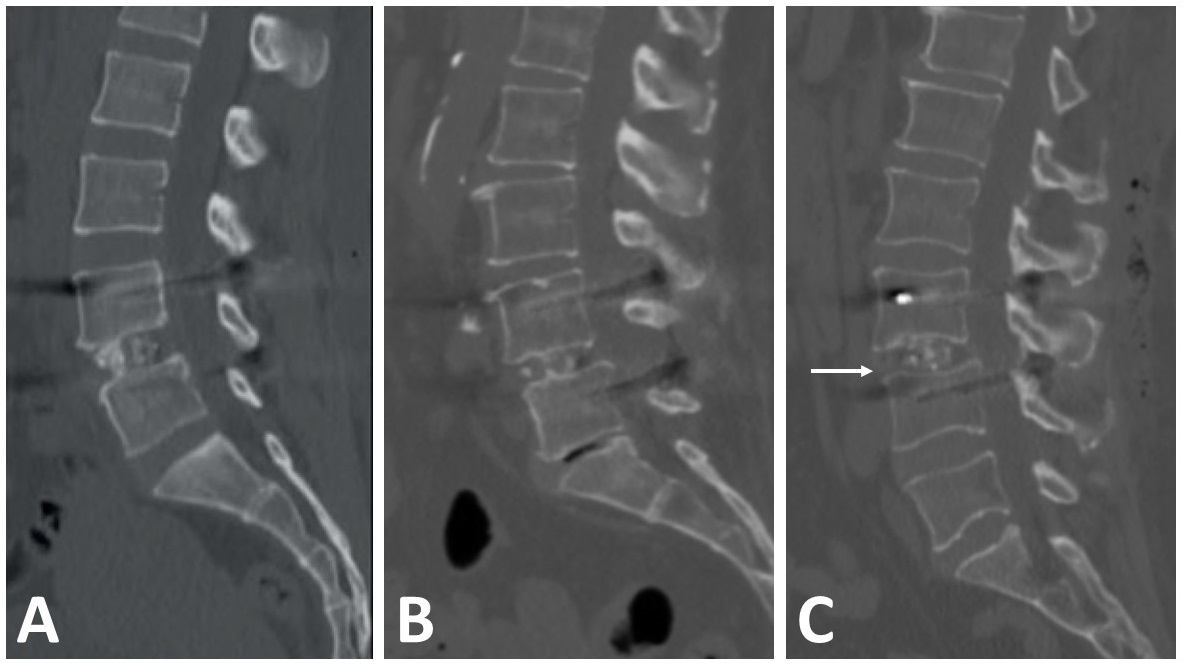

Supplement: Supplementary file 1 — Supplementary Fig. 1: Representative examples of fusion grades according to the Bridwell grading system. A Grade I: Solid fusion with trabecular continuity and remodeling. B Grade II: Intact graft with incomplete remodeling and no radiolucent lines. C Grade III: Potential radiolucency at the graft–endplate interface (white arrow). Grade IV (graft collapse or resorption indicating nonunion) was not observed in our series [file 13018_2026_6946_MOESM1_ESM.jpg]
